# Next-generation protein-based materials capture and preserve projectiles from supersonic impacts

**DOI:** 10.1038/s41565-023-01431-1

**Published:** 2023-07-03

**Authors:** Jack A. Doolan, Luke S. Alesbrook, Karen Baker, Ian R. Brown, George T. Williams, Kira L. F. Hilton, Makoto Tabata, Penelope J. Wozniakiewicz, Jennifer R. Hiscock, Benjamin T. Goult

**Affiliations:** 1 School of Biosciences, University of Kent, Canterbury, UK; 2 School of Chemistry and Forensic Science, University of Kent, Canterbury, UK; 3 Department of Physics, Chiba University, Chiba, Japan; 4 School of Physics and Astronomy, University of Kent, Canterbury, UK; 5 Present Address: Department of Chemistry, University of Southampton, Southampton, UK

**Keywords:** Biomaterials, Biomaterials, Polymer chemistry

## Abstract

Extreme energy-dissipating materials are essential for a range of applications. The military and police force require ballistic armour to ensure the safety of their personnel, while the aerospace industry requires materials that enable the capture, preservation and study of hypervelocity projectiles. However, current industry standards display at least one inherent limitation, such as weight, breathability, stiffness, durability and failure to preserve captured projectiles. To resolve these limitations, we have turned to nature, using proteins that have evolved over millennia to enable effective energy dissipation. Specifically, a recombinant form of the mechanosensitive protein talin was incorporated into a monomeric unit and crosslinked, resulting in a talin shock-absorbing material (TSAM). When subjected to 1.5 km s^−1^ supersonic shots, TSAMs were shown to absorb the impact and capture and preserve the projectile.

## Abstract (web summary)

An engineered version of the mechanosensitive protein talin was used as a monomer in combination with a synthetic chemical crosslinker to form a hydrogel. This shock-absorbing material is shown to capture and preserve projectiles fired at 1.5 km s^−1^.

## Main

When impacted by a projectile, a material is exposed to various phenomena simultaneously. To survive the impact, a material must contend with wave propagation (elastic, shock and plastic), fragmentation, perforation and spallation^1^. Thus, installing a mechanism within a material to enable effective energy dissipation is essential for multiple applications^2–4^. Military and civilian forces commonly use body armour to protect the wearer against penetration from projectiles, such as bullets or shrapnel^4^. Frequently, this armour consists of a multilayered system, commonly a ceramic face backed by a fibre-reinforced composite^5^. This multilayered design enables the hard, brittle ceramic to destroy the projectile tip, distributing the kinetic energy over the backing, which reflects the tensile wave and captures the shattered ceramic^6^. Despite the effective penetration blocking of these armour systems, a remainder of the kinetic energy is still distributed to the wearer, often resulting in behind-armour blunt trauma^7^. Furthermore, during impacts, this form of armour is irreversibly damaged, compromising its structural integrity for further use. The aerospace sector uses impact energy-dissipating materials for the unique task of capturing and preservation of space debris, space dust and micrometeoroids^8^. These captured projectiles contribute towards our understanding of the local environments of aerospace equipment, including that of the International Space Station^9^. Data from these experiments facilitate aerospace equipment design, improving the safety of astronauts and the longevity of costly aerospace equipment. Aerogels are the current industry standard for projectile capture and preservation, achieving energy dissipation through the conversion of projectile kinetic energy into both mechanical and thermal energy^10^. However, the resulting temperature elevation, further enhanced by the remarkable insulating properties of aerogel^11^, can cause the aerogel structure to melt^10^. Furthermore, these elevated temperatures may compromise the structure of the captured projectiles, altering their chemical composition^10,12^. This thermal and mechanical energy causes chemical bond breakage, rendering the aerogel irreversibly damaged postimpact. It is apparent from the aforementioned examples that a material using an energy dissipation mechanism that reforms following the removal of force would alleviate inherent issues seen with the industry standard materials. Additionally, specifically for the aerospace sector, energy dissipation that does not result in the conversion of kinetic to thermal energy would be beneficial.

**Fig. 1:**
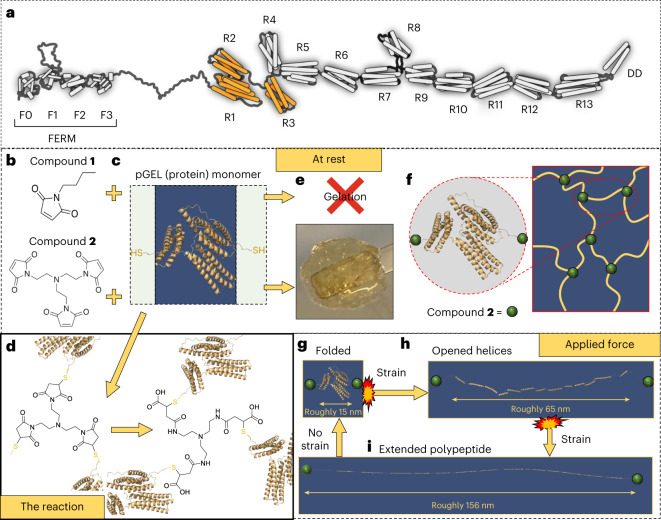
The design concept of TSAM. **a**, Cartoon representation of the protein talin. F, FERM domain; R, rod domain; DD, dimerization domain^20^. The R1−R3 domains that were engineered to form the pGEL monomer are highlighted in orange. **b**, Chemical structure of the control compound **1** and the trivalent crosslinker **2**. **c**, pGEL in the folded state: green boxes show the flexible linkers with a terminal thiol containing cysteine residue and the blue box shows modified R1–R3 domains of talin. **d**, Reaction of the pGEL monomer with compound **2** (not to scale). **e**. Hydrogel formed from the reaction of the pGEL monomer (200 mg ml^−1^) with **2**. No gelation was observed with **1**. **f**, The proposed network structure formed at the molecular level through the reaction of thiol containing cysteine residues within the pGEL monomer and **2** with no applied strain. **g**, pGEL in a fully folded state presents length of roughly 15 nm, measurements based on the estimated length of R1–R3 of the wild-type protein in resting state^31,32^. **h**, When exposed to strain, pGEL unfolds into a linear string of helices extending to roughly 65 nm in length, measurements based on estimated length of R1–R3 of the wild-type protein^18,26,31^. **i**, When exposed to higher strain, pGEL unfolds fully into an extended polypeptide, increasing to a length of 156 nm, measurements based on estimated length of R1–R3 of the wild-type protein under more than 25 pN (ref. ^26^). The R1–R3 rod domains refold once the strain is removed^26^.

Within the animal kingdom, proteins that offer unique mechanical properties are rife; silk fibroin displays modifiable macroscale properties in its assembled fibre form, while elastin instils elasticity in animal tissues^13^. Although there are many proteins analogous to these examples, very few researchers have tapped into these natural resources for development of materials with enhanced mechanical properties^14–16^; even fewer have tested these materials for applications outside the biomedical sector^13^. Talin (Fig. 1a) is the epitome of a mechanical protein, mediating the connection between the actin cytoskeleton and the integrin extracellular matrix receptors, acting as a mechanosensor. Previous work determined that, through unfolding and refolding events of its 13 four- or five-helical rod domains^17–20^ when stretched within the physiologically relevant range, talin is able to maintain the average force experienced by the protein below 10 pN (ref. ^18^). Furthermore, on removal of force, refolding of the talin rod domains occurs with high fidelity over numerous force cycles^18^ confirming talin to be a cellular shock absorber.

Here, we have engineered a recombinant form of talin, termed pGEL, which comprises three rod domains of talin, R1–R3, that are modified (with internal cysteine residues mutated to serine and cysteine residues introduced at either end of the protein) for use as the monomer with which to form a polymer. When exposed to force, these three domains provide a stepwise unfolding, with the wild-type domains exhibiting threshold unfolding forces of 20, 15 and 5 pN, respectively^18^. We proposed that, on application of force (that is, shear strain or impact), the three rod domains within each protein monomer would unfold, dissipating energy through the endothermic process of protein unfolding^21^ (Fig. 1g–i).

Using compounds **1** (control monovalent compound) and **2** (trivalent crosslinker) (Fig. 1b and Supplementary Fig. 1a–d), pGEL (Fig. 1c) was formed into a hydrogel (Fig. 1e,f) via tri-substitution of the terminal cysteines with crosslinker **2** (Fig. 1d and Supplementary Fig. 1e–g). The resulting hydrogel, which we have termed TSAM (talin shock-absorbing material), therefore, contains monomeric units capable of refolding on removal of force, retaining its energy-dissipating mechanism following any potential impact events. Due to the endothermic energy-dissipating mechanisms of protein unfolding^21^ in TSAM, the heating of the captured projectiles seen with the aerogel energy-dissipating mechanism would not be observed, offering a solution to several of the limitations seen with current state-of-the-art impact absorption materials. The use of compound **1** supports our conclusion that compound **2** is responsible for the pGEL monomer polymerization processes and resultant material formation (Fig. 1d).

## TSAM structural characterization

**Fig. 2:**
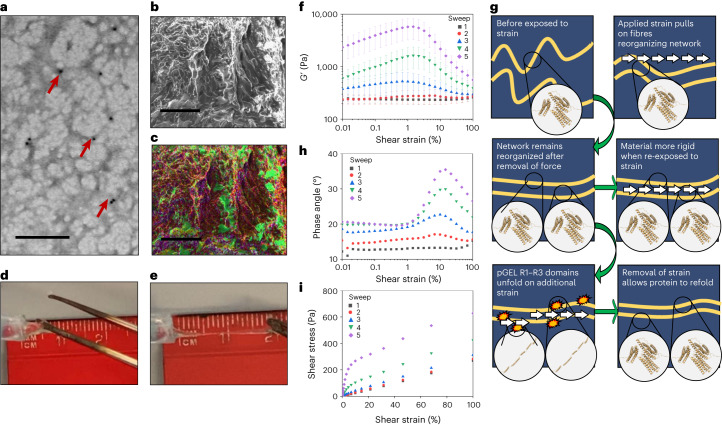
The internal fibre structure of TSAM and its macroscale characterization. **a**, Immuno-gold-stained TSAM imaged with TEM showing lattice structure of connected pGEL proteins. Gold particles are observed as black dots, some of which are highlighted with red arrows. Scale bar, 200 nm. **b**, The dense fibre structure of TSAM displaying a porous network imaged with SEM on secondary electron mode. Scale bar, 50 µm. Pore sizes are in the range of a few micrometres. **c**, EDX analysis of SEM image in **b**. Sulfur, yellow; carbon, red; oxygen, green; sodium, teal; phosphorus, purple. Scale bar, 50 µm. Please note that SEM and the TEM show the overall topology of the gel at two different resolution scales. **d**, TSAM is slightly stretched. **e**, TSAM stretched to three times its length. **f**–**i**, Rheological measurements of TSAM (*n* = 3). **f**, *G*’ as a product of shear strain (error bars, SEM) for sweeps 1 (squares), 2 (circles), 3 (triangles), 4 (inverted triangles) and 5 (diamonds). **g**, Schematic summary of the events proposed to occur over five repeated oscillatory sweeps. **h**, Phase angle against shear strain for sweeps 1–5 on TSAM. **i**. Shear stress against shear strain for sweeps 1–5 on TSAM. Data points are from five technical repeats on each sample with a 2 min delay between each, and error bars are from three technical repeats (SEM). Source data

The R1–R3 domains of talin incorporated in the pGEL monomer were confirmed to retain alpha-helical folding using circular dichroism and ^1^H-^15^N heteronuclear single quantum coherence nuclear magnetic resonance (Supplementary Fig. 2). Following the formation of TSAM, the characterization of the internal network structure was conducted. Fourier transform infrared spectroscopy^22^ confirmed that the helical nature of the talin domains was still present in the material structure (Supplementary Fig. 3). His-tagged gold immunostaining of the TSAM, imaged using transmission electron microscopy (TEM), confirmed the presence of pGEL in a lattice formation, displaying pore sizes of approximately 100 nm (Fig. 2a). Following this, scanning electron microscopy (SEM) revealed TSAM to contain a porous-like structure on the micrometre scale typical of hydrogels (Fig. 2b), with long fibres roughly 2 µm wide and pores of roughly 10 µm in diameter. Energy dispersive X-ray (EDX) analysis confirmed that the observed fibres in the SEM images consisted of sulfur and carbon (Fig. 2c), pGEL representing the only component of the xerogel containing these atoms. Together, these findings indicated pGEL molecules linked with crosslinker **2** form a lattice on the nanometre scale, morphing into larger fibrillar-like structures on the micrometre scale. When handling TSAMs, high levels of extensibility were observed, presenting an extension of more than threefold when under tension and returning to the original size on force removal (Fig. 2d,e).

## Evidence for pGEL domain unfolding in TSAM

Rheological characterization of TSAMs provided strong evidence for the induced unfolding of the talin domains within the material when exposed to shear strain, indicating that the energy-dissipating mechanisms of talin were successfully incorporated into the TSAM. Here, oscillatory shear strain sweeps were conducted. A sinusoidal oscillation of a preset shear strain was applied to the TSAM at a set frequency, with the resultant shear stress measured. A total of five consecutive oscillatory shear strain sweeps, with a 2-minute rest period between each sweep, were conducted on three different TSAM samples to ensure the reproducibility of results.

For the first applied oscillatory shear strain sweep on the TSAM, the dynamic shear storage (*G*’) and loss modulus (*G*”), as a product of shear strain, presented a linear viscoelastic region (LVER) for the full range of shear strain tested (Supplementary Fig. 4a,b). The LVER indicates the degree of shear strain at which the material acts elastically^23^, revealing the TSAMs as durable materials with high levels of extensibility. Following this, four further oscillatory shear strain sweeps were conducted (Supplementary Fig. 4a–j and Fig. 2f). Owing to the unfolding and refolding kinetics intrinsic to R1–R3, we proposed viscoelastic properties would be retained on repeated exposure to shear strain. As proposed, during all five oscillatory shear strain sweeps, the TSAMs presented *G*’ > *G*” throughout the range of shear strain tested, confirming conserved viscoelastic behaviour (Supplementary Fig. 4). During sweeps 3–5, a positive gradient of *G*’ occurring with increased shear strain on the *x* axis (Fig. 2f) was observed in place of the LVER seen with sweeps 1 and 2. Such an observation is termed strain stiffening. Here, the peak maxima for *G*’ occurred between 1 and 5% for sweeps 3–5, shifting to the right and increasing in amplitude for each subsequent sweep (Fig. 2f). Strain stiffening was further observed as the concomitant increase of the complex modulus (G*) (sum of *G*’ and *G*”) with accumulated sweeps. The strain stiffening across and between sweeps reveals TSAMs to present increased resistance to deformation on repeated exposure to shear strain^24^. Strain stiffening due to fibre reorganization, such as the TSAM fibres depicted in Fig. 2b, is a well-documented phenomenon occurring in hydrogels formed from biopolymers^25^, causing the elastic modulus to increase with strain. We propose that the strain stiffening observed here results from a greater number of talin domains arranged in parallel to the axis of the fibres (Fig. 2g), resulting in the overall increased network rigidity observed.

Following the peak maxima of *G*’ for sweeps 3–5 displayed in Fig. 2f, *G*’ decreased with increasing shear strain. A decrease in *G*’ indicates a reduction in the rigidity of the material. Furthermore, the phase angle across each sweep (displayed in Fig. 2h) revealed a bell-shaped curve, with the peak amplitude of the bell increasing for each subsequent sweep. The positive slope of the phase angle in Fig. 2h corresponded to the negative gradient of *G*’ in Fig. 2f for the same respective sweep. Thus, an increase in phase angle was observed simultaneously with a decrease in rigidity. A sudden increase in phase angle is caused by a lag between the applied sinusoidal shear strain and the resulting shear stress, occurring from a rapid increase in viscosity. The phase angle reached a maximum at roughly 10–15% shear strain, with the phase angle then declining, revealing a decrease in viscosity with the further increase of shear strain. When combined, the above observations were all accounted for by the induction of the pGEL monomer unfolding within TSAMs (Fig. 2g). Specifically, the increased network rigidity means strain can be imparted on the fibres themselves. When a maximum fibre strain is reached, mass chain unfolding of the TSAM R1–R3 domains occurs, reducing the rigidity of the material and introducing slack into the system from the extension (Fig. 1g–i) of the now unfolded pGEL monomer domains. This slack registers as the decreased rigidity (decrease in *G*’) and sudden increase in phase angle. Following the application of increasing shear strain, the slack from unfolded TSAM R1–R3 domains is taken up, increasing the tension on the fibres and, as a result, the material’s rigidity. This is observed as the decrease in phase angle (Fig. 2h) and increase in *G*’ (Fig. 2f). On the other hand, if the material were reaching a gelation to solution transition point as a consequence of internal structure breakage, the phase angle would have continued to increase above 45°. On removal of shear strain, the unfolded R1–R3 domains may then refold, and the resulting TSAM displays an enhanced rigidity (higher G* at the start of the next sweep) due to fibre network reorganization. Shear stress versus shear strain correlations corroborate these results, revealing an exponential increase in shear modulus (*G*), a measure of rigidity, with accumulated sweeps, further illustrating the strain stiffening within the TSAM (Fig. 2i). Furthermore, sweeps 4 and 5 reach shear yield points, beginning to move into viscous stress as seen by the induction of a slope, subsequently transitioning back into a linear gradient indicating the reoccurrence of elastic behaviour. In summary, the linear elastic region at low shear strain is a result of reordering of the network structure and gradual tension accumulating in the fibres; the following curve transition indicates the sudden mass unfolding of talin rod domains, and subsequent linear region reports elasticity reoccurring once tension is again applied to the fibres with increasing shear strain.

**Fig. 3:**
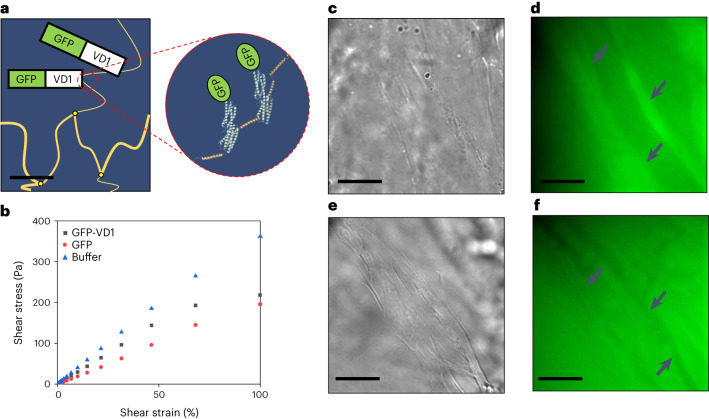
Effects of GFP-VD1 on TSAM. **a**, Representation of GFP-VD1 binding to unfolded pGEL in TSAM fibres, with resulting cartoon protein figures created in PyMOL using VD1 Protein Data Base (PDB) structure 1U6H (ref. ^33^). Scale bar, 100 nM. **b**, Shear stress as a product of shear strain for buffer (blue triangles), GFP-VD1 (black squares) and GFP (red circles), showing GFP-VD1 treated TSAM reaches its yield point between 46 and 68% shear strain. **c**, Transmitted light image of GFP-VD1 localized to TSAM fibres. Scale bar, 20 µm. **d**, Maximum projection widefield fluorescent image of **c** (scale bar, 20 µm) with fibres showing localized GFP-VD1 indicated by arrows. **e**, Transmitted light image of GFP in TSAM. Scale bar, 20 µm. **f**, Maximum projection widefield fluorescent image of **e**, showing GFP sitting in void space, with fibres this time visible as darker structures indicated by arrows. Scale bar, 20 µm. Source data

To confirm that the unfolding of the talin domains within the TSAM was directly responsible for the rheological characteristics and material properties observed, a green fluorescent protein tagged-vinculin domain 1 protein (GFP-VD1) was used. GFP-VD1 can selectively bind to the unfolded state of each rod domain (Fig. 3a and Supplementary Fig. 5a), preventing domain refolding and ‘locking’ the extended conformation^26^. Here the GFP-VD1 was introduced into the TSAM pre-amplitude sweep as a 2 mg ml^−1^ solution through a material swelling process. The rheological properties of these materials were then explored and compared to the results of analogous studies in which the same TSAM material underwent the same material swelling process in a solution of GFP or buffer only. The resulting *G*’ and *G*” as a product of shear strain for the three conditions tested are summarized in Supplementary Fig. 5b,c.

When plotted as shear stress against shear strain (Fig. 3b), the GFP and buffer controls presented the same linear trend as obtained in the first amplitude sweep for the non-treated TSAMs, indicating purely elastic behaviour. By contrast, the TSAM treated with GFP-VD1 reached a yield point between 46 and 68% shear strain (Fig. 3b) due to VD1 binding events. A series of comparative fluorescence microscopy experiments were conducted to further confirm the binding of GFP-VD1 to the TSAM fibres. Here, fibre-like structures exhibiting the same diameter as those observed in our previous SEM studies (Fig. 2b) were found to have localized GFP-VD1 (Fig. 3c,d), confirming binding. In contrast, the GFP control-treated TSAM fibres appeared as darker regions, with void spaces presenting higher GFP concentrations (Fig. 3e,f).

## TSAMs capture and preserve supersonic projectile impacts

Following the rheological evidence for TSAM’s retention of talin’s endothermic energy dissipation mechanism, we moved on to test the performance of the TSAM as an impact-absorbing material, investigating TSAM performance on supersonic projectile impact. Specifically, velocities of 1.5 km s^−1^ were tested, as this is a speed relevant to the aerospace and defence industries^27,28^. For instance, particles in space impact both natural and human-made objects at speeds greater than 1 km s^−1^ (ref. ^27^). In contrast, muzzle velocities from firearms commonly fall between 0.4 and 1.0 km s^−1^ (ref. ^28^). Here the TSAMs, in addition to a commercially available polyvinylpyrrolidone hydrogel control, were placed in the target chamber of a light gas gun (LGG) and the following material properties tested: (1) the ability of the TSAM to survive impact; (2) the ability of the TSAM to reduce the force of the projectile before impacting an aluminium back plate and (3) the ability of the TSAM to capture the projectile in a preserved state.

**Fig. 4:**
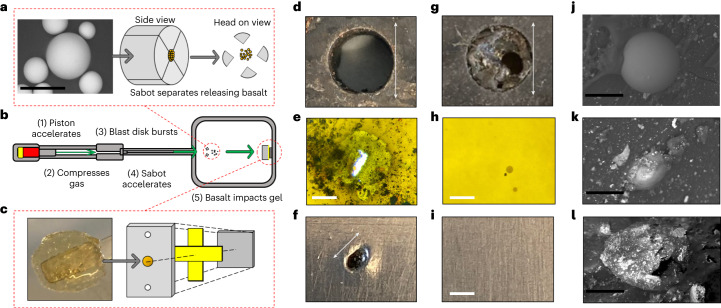
Supersonic impact study on TSAM. **a**, SEM image of a basalt particle used as the projectile and representation of how it is loaded into a sabot and released during a shot. Scale bar, 60 µm. **b**, Diagram of the LGG apparatus with the key stages after the shot is triggered. **c**, Image of TSAM and how it is prepared as a target. The TSAM is loaded into a target plate constructed of steel (BTEA, stainless 304), with tape used to seal the back of the hole, followed by an aluminium back plate (Al 5083). **d**–**f**, Results from control gel. **d**, Destroyed control gel after basalt impact at 1.5 km s^−1^. Scale bar, 8 mm. **e**, Hole formed in tape from basalt projectile. Scale bar, 1 mm. **f**, Crater formed in aluminium back plate. Scale bar, 1.33 mm. **g**–**i**, The results from TSAM. **g**, Mostly intact TSAM after basalt impact at 1.5 km s^−1^. Scale bar, 8 mm. **h**, Tape with no hole, containing several caught basalt particles in the transparent TSAM attached to its surface (Supplementary Fig. 6). **i**, Undamaged aluminium back plate. Scale bars, 1 mm. **j**, SEM image of intact basalt particle caught by TSAM after impact at 1.5 km s^−1^. Scale bar, 45 µm. **k**, SEM image of another basalt particle caught by TSAM after impact at 1.5 km s^−1^. Scale bar, 30 µm. **l**, SEM image of a fragment of the aluminium (Al 7075) burst disc that impacted TSAM during the 1.5 km s^−1^ basalt shot. Scale bar, 50 µm. Results from repeat experiments and further images can be found in Supplementary Figs. 6 and 7.

Spherical basaltic particles between 20 and 70 µm were used as projectiles, loaded in a sabot as buckshot. A schematic for this experiment is given in Fig. 4a–c. When shot at 1.5 km s^−1^, the upper ballistics limit for terrestrial-based weaponry and non-meteorite impact, the control gel was destroyed (Fig. 4d), with a visible hole in the tape behind the gel (Fig. 4e), and a crater of 1.33 mm in diameter produced in the aluminium back plate (Fig. 4f). Therefore, this material control showed no detectable impact absorption properties. However, under analogous experimental conditions, the TSAM appeared primarily intact from the frontal perspective (Fig. 4g and Supplementary Fig. 6), with no projectile permeation detected to either the supporting tape (Fig. 4h) or the aluminium back plate (Fig. 4i). In addition, subsequent SEM analysis identified the basalt particles embedded in the TSAM post shot (Fig. 4j and Supplementary Fig. 7), confirming that the TSAM had wholly absorbed the impact of the basalt buckshot. An equivalent shot into aerogel revealed most basalt particles penetrated between 5 and 8 mm (Supplementary Fig. 8) indicating TSAM is competitive in performance with this industrial standard, as the TSAM protected the material backing from any impact damage with 5 mm depth of material. The transparency of the TSAM shown in Fig. 4c and Supplementary Fig. 7 is an additional desirable property, allowing for the easy removal of caught projectiles from the TSAMs. To conclusively determine whether TSAM also enabled the preservation of the captured basalt projectiles, SEM was performed on the impacted TSAM. Multiple basalt particles presenting a preserved circular shape were observed in the gel (Fig. 4j,k), confirmed as basalt with EDX analysis (Supplementary Fig. 9). Thus, supporting that TSAM enables projectile preservation. Moreover, during one of the TSAM shots, shrapnel from the aluminium (Al 7075) burst disc (Fig. 4b) struck the TSAM in combination with the basalt, as confirmed through SEM and EDX analysis (Fig. 4l and Supplementary Fig. 10). Such an impact often destroys aerogel materials used as the industry standard within the aerospace industry for projectile capture, providing evidence that TSAMs are able to overcome this limitation.

## Conclusions

We present TSAM, a SynBio material constructed from monomeric units containing force-dependent mechanical switch domains. Our previous work demonstrated that multiple talin domains in series enable talin to serve as a force buffer during large strain changes^18^. The TSAM material was designed to capture this shock-absorbing property of the monomers on a macroscale. In addition, we show that TSAMs can absorb supersonic projectile impacts by basalt particles and larger pieces of aluminium shrapnel. Hydrogels produced from gelatine and recombinant telechelic proteins have been used in impact studies previously^29^, but these lack the intrinsic mechanosensitive properties of talin and therefore also the pGEL monomer. The difference between the TSAM and gelatine systems is further supported when considering work by Kokol and coworkers^30^. Here gelatine hydrogels demonstrate notable variations in material characteristics compared to the TSAM systems. These variations in material characteristics will result in differences in material properties and response to impact. These results lend the TSAMs towards application within the aerospace and defence industries, for example as a backing for multilayered armour where shattered ceramic capture is required, and in hypervelocity impact experiments in which the projectile needs to be preserved for further study. We believe this is due to the endothermic energy-dissipating mechanism of talin^21^. This energy-dissipating mechanism was confirmed using rheology, while GFP-VD1 binding experiments supported the presence of talin unfolding events within these processes. Furthermore, through the reversible refolding of talin domains within TSAM following the removal of the force, the material also demonstrates potential for iterative use. As talin contains 13 helical domains, each with unique unfolding forces, these TSAMs may be tuneable by modifying the talin domains featured in the monomer unit, offering the potential for tailoring toward a diverse array of mechanical properties and resulting applications.

## Methods

### Protein engineering

The genes encoding pGEL, GFP-VD1 and GFP were constructed in pET151 vectors. The proteins were expressed in BL21(DE3)* *Escherichia* *coli*. Protein purification was achieved using HisTrap HP columns (Cytiva) for His-tag based affinity chromatography using an AKTA Start protein purification system (Cytiva). Following purification, proteins were dialysed in phosphate buffer (20 mM sodium phosphate, pH 7.4, 50 mM NaCl).

### TSAM preparation

Here, TCEP (tris(2-carboxyethyl)phosphine) is used as a reducing agent. A 30:1 ratio of TCEP:cysteine was slowly added to a solution of pGEL (200 mg ml^−1^) in phosphate buffer (pH 7.4). After 1 h, the pGEL solution was run through PD10 desalting columns (Cytiva) twice to ensure TCEP removal. Immediately following the desalting step, the pGEL solution was concentrated to the desired concentration using 30 kDa molecular weight cut-off concentrators (SigmaAldrich). The TSAM was then formed through the addition of crosslinker **2** at 1:1 maleimide:thiol molar ratio, which is equivalent to a 2:3 crosslinker **2**:pGEL monomer molar ratio. The TSAM was left to set at 4 °C overnight.

### SEM

The TSAM sample was placed into a petri dish and left at 37 °C until the material had completely desiccated, producing a xerogel. This xerogel was then placed on a carbon tab mounted onto an aluminium stub. Imaging was achieved using a Hitachi S-3400N scanning electron microscope with EDX analysis and analysed using Oxford Instruments AZtec software.

### Immuno-gold staining and TEM

A 2 µl amount of sample was applied to carbon and formvar 400 mesh gold grids (Agar Scientific) and allowed to settle on the grid for 5 min. The sample was then fixed in 2% formaldehyde and 0.5% glutaraldehyde in 100 mM sodium cacodylate buffer pH 7.2 for 15 min at room temperature. Samples were washed 2 × 5 min in cacodylate buffer and 2 × 5 min in 20 mM Tris, 500 mM NaCl, 0.1% BSA and 0.5% Tween 20 (TBST). Grids were blocked in 2% BSA in TBST for 30 min and then moved into a 20 µl drop of anti-His-tag primary antibody (Sigma) diluted 1:100. Grids were washed 6 × 2 min in drops of TBST before incubation in Goat anti-mouse IgG conjugated to 5 nm gold particles (British Biocell International) diluted 1:50 for 30 min. Grids were washed for 6 × 2 min in TBST and 6 × 2 min in distilled water. Negative controls were performed as above but primary antibody was replaced with TBST. Samples were then air dried and negative stained in 2% aqueous uranyl acetate. Samples were viewed using a Jeol 1230 transmission electron microscope at 80 kV and images were recorded on a Gatan OneView 16 megapixel digital camera.

### Rheological measurements

Rheological measurements were performed on an Anton Parr modular compact rheometer (MCR302). All measurements were performed at 298 K using a PP20 parallel plate. Oscillatory amplitude experiments maintained a frequency of 10 rad s^−1^ and were performed with an amplitude of oscillation range of 0.01–100%. A 2 min rest time was set between each amplitude sweep, with a total of five sweeps performed on each TSAM. For the GFP-VD1, GFP and buffer swelled experiments, the TSAM was left in 2 mg ml^−1^ of the respective solution overnight before rheological measurements were performed. For the GFP-VD1 experiments, TSAMs were swelled with GFP, GFP-VD1 or buffer by being placed in the respective solution overnight at 4 °C.

### Fluorescence microscopy

Following rheology experiments, the resulting GFP and GFP-VD1 swelled TSAMs were washed overnight at 4 °C in solution of buffer to reduce background fluorescence from unbound protein. GFP-VD1 and GFP treated samples of TSAM from the rheology experiments were visualized using an Olympus IX71 microscope using a ×1.6 magnification Optovar in combination with a PlanApo ×100 OTIRFM-SP 1.49 numerical aperture lens mounted on a PIFOC *z* axis focus drive (Physik Instrumente), and illuminated using LED light sources (Cairn Research Ltd) with DC/ET350/50x excitation, ET Quad Sedat dichroic and DC/457/50m emission filters (Chroma). Samples were visualized using a QuantEM (Photometrics) EMCCD camera, and the system was controlled with MetaMorph software (Molecular Devices). Each three-dimensional maximum projection of volume data was calculated from 31 *z* plane images and the best six were chosen, each 0.2 µm apart, and analysed using MetaMorph software.

### LGG experiments

The impact experiments were carried out using the LGG facility at the University of Kent, Canterbury. The LGG is capable of accelerating projectiles smaller than 3.5 mm to speeds up to 7 km s^−1^ (refs. ^34,35^). The TSAM or control target (8 mm diameter and 5 mm thick, chosen on the basis of sample holder size) was set in a blast tank exit aperture (BTEA) with a circular, 8 mm diameter aperture, sealed with tape, with an aluminium (5083) back plate placed behind. Basalt particles (20–70 μm) were loaded into a single sabot using the ‘buckshot’ method and were fired at roughly 1.5 km s^−1^, with the speeds recorded via the BTEA-muzzle laser method as described by Burchell et al.^34^. The target was removed before the air flushing procedure to reduce gun contamination of the TSAM. The combination of the BTEA and target mount into a single device allowed for minimal spreading of the buckshot projectile, increasing the chance of direct impact onto the TSAM and maximizing the BTEA-muzzle separation. Discussion about talin unfolding rates is provided within the Supplementary Information.

### Aerogel experiments

For comparison to the industry standard material, a LGG shot on aerogel was performed. The block of aerogel displayed an initial manufacturing density of 0.092 g per cc and measured 30 × 30 × 20 mm. Before setup, the aerogel was baked overnight to remove any built-up moisture in the block and reweighed. The post baking density was found to be 0.09 ± 0.01 g per cc. The block was positioned in the blast tank of the LGG and backed with an aluminium target plate (Supplementary Fig. 20a). The projectile was prepared in exactly the same way as the TSAM and control gel shots, and fired at approximately 1.5 km s^−1^. The projectile consisted of a 0.170 sabot with an internal diameter bore of 0.8 mm loaded with basalt spheres of sizes 20–70 µm. Microscopy images were captured using a Leica PLANAPO ×1.0 microscope and images were processed using Leica Application Suite X (v.3.7.5.24914).

### Statistics and reproducibility

All rheology and impact experiments conducted with the control and TSAM materials were repeated for three different samples to ensure the results presented are robust and reproducible. No data were excluded from analysis.

## Online content

Any methods, additional references, Nature Portfolio reporting summaries, source data, extended data, supplementary information, acknowledgements, peer review information; details of author contributions and competing interests; and statements of data and code availability are available at 10.1038/s41565-023-01431-1.

## Supplementary information

Supplementary Information Supplementary Methods and Figs. 1–10.

## Data Availability

Any data are available from the corresponding authors upon reasonable request. Source data are provided with this paper.

## Source data

Source Data Fig. 2f,h,i Source data from Rheometer for Fig. 2f. Source data from Rheometer for Fig. 2h. Source data from Rheometer for Fig. 2i.
Source Data Fig. 3b Source data from Rheometer for Fig. 3b.
