# Supplementary material for: Next-generation protein-based materials capture and preserve projectiles from supersonic impacts
Source: Nat Nanotechnol. 2023 Jul 3;18(9):1060–6. doi: 10.1038/s41565-023-01431-1 (PMC10501900; doi:10.1038/s41565-023-01431-1)
Supplement: Supplementary file 1 — Supplementary Methods and Figs. 1–10. [file 41565_2023_1431_MOESM1_ESM.pdf]

nature nanotechnology

Article

https://doi.org/10.1038/s41565-023-01431-1

# Next-generation protein-based materials capture and preserve projectiles from supersonic impacts

In the format provided by the
authors and unedited

***Supplementary methods*** ........................................................................................ **2**

***Supplementary Figures*** ........................................................................................ **5**

Supplementary Fig. 1 Compound characterisation. ........................................................................5
Supplementary Fig. 2 pGEL characterisation. ..............................................................................6
Supplementary Fig. 3 TSAM characterisation. ..............................................................................7
Supplementary Fig. 4 TSAM rheology. Rheology data for five amplitude sweeps. a-b. ................8
Supplementary Fig. 5 Analysis of TSAM with GFP-VD1, GFP and buffer. ...................................9
Supplementary Fig. 6 Supporting images from the Light Gas Gun (LGG) experiments .............10
Supplementary Fig. 7 Supporting images from the Light Gas Gun (LGG) experiments .............11
Supplementary Fig. 8 Supporting images from the Light Gas Gun (LGG) experiments using Aerogel. ..................................................................................................................................12
Supplementary Fig. 9 EDX analysis of caught basalt particles from light gas gun experiments imaged in SEM. .......................................................................................................................13
Supplementary Fig. 10 EDX analysis of caught burst disc shrapnel in light gas gun experiment imaged with SEM. ...................................................................................................................14

***Methods-only references*** ........................................................................................ **15**

## Supplementary methods

**Crosslinker 1 synthesis:** This compound was synthesised as described by Eloh *et al.*[36] with minor modifications. Maleic anhydride (1.00 g, 10.00 mmol) was dissolved in dichloromethane (DCM) (15.00 mL). *N*-butylamine (1.00 mL, 1.00 mmol) was added and the mixture was stirred at room temperature for 1 hour. The solvent was removed *in vacuo*, and the resulting white powder was re-dissolved in acetic anhydride (6.00 mL). To this solution, sodium acetate (0.50 g, 6.10 mmol) was added, and the mixture was heated at 80°C under reflux for 2 hours. The solution was diluted with distilled water (50.00 mL) and washed with diethyl ether (3 x 50.00 mL). The organic layer was collected and further washed with 0.1 M hydrochloric acid (1 x 50.00 mL) and 0.1 M sodium hydroxide (1 x 50.00 mL). The organic layer was dried over anhydrous sodium sulphate, filtered, and concentrated *in vacuo* to give the crude product as a colourless liquid. The N-butylmaleimide was further purified using silica chromatography, 85:15 (Ethyl acetate:hexane), producing a yellow oil with a yield of 11% (0.17 g, 11.00 mM). $^1$H NMR (400 MHz, 298 K, DMSO-$d_6$): δ: 7.01 (s, 2H), 3.38 (t, *J* = 7.06 Hz, 2H), 1.46 (m, 2H), 1.21 (m, 2H), 0.86 (t, *J* = 7.36 Hz, 3H) data was consistent with previously reported values.

**Crosslinker 2 synthesis:** This compound was synthesised as described by Hanlon *et al.*[37] with minor modifications. A solution of maleic anhydride (0.59 g, 6.00 mmol) in anhydrous dimethyl formamide (DMF) (2.43 mL) was prepared under inert atmosphere and cooled to 0°C. Separately, a solution of tris(2-aminoethyl)amine (0.29 mL) in dry DMF (2.04 mL) was prepared under inert atmosphere, and added dropwise to the maleic anhydride solution at 0°C over 30 minutes. The solution was stirred for a further 30 minutes at 0°C. A solution of sodium acetate (0.048 g, 0.60 mmol) in acetic anhydride (0.60 mL) was added to the reaction mixture at room temperature and stirred overnight at 50°C under inert atmosphere. The reaction mixture was concentrated using rotary evaporation, resuspended in DCM (50.00 mL) and washed with saturated brine (6 x 50.00 mL). The organic layer was collected, concentrated using rotary evaporation, resuspended in DCM (50.00 mL) and further washed with saturated sodium bicarbonate solution (6 x 50.00 mL). The organic layer was collected and concentrated using rotary evaporation to obtain the crude product. The crude product was purified using silica chromatography, 85:15 (Ethyl acetate:hexane). The resulting pure yellow crystalline product was dried under vacuum overnight with a yield of 9% (0.21 g, 0.54 mM). $^1$H NMR (400 MHz, 298 K, DMSO-$d_6$): δ: 6.98 (s, 6H), 3.38 (t, *J* = 6.60 Hz, 6H), 2.60 (t, *J* = 6.62 Hz, 6H) data was consistent with previously reported values.

**Compound NMR characterisation:** NMR spectra for compounds **1** and **2** were obtained on a Bruker AV2 400 MHz spectrometer. The data was processed using TopSpin. NMR chemical shift values are reported in parts per million (ppm) and calibrated to the centre of the residual solvent peak set (s = singlet, br = broad, d = doublet, t = triplet, q = quartet, m = multiplet).

**Electrospray Ionisation mass spectrometry:** ESI-MS was performed on an Agilent HPLC system connected to a Bruker micrOTOF-Q mass spectrum instrument. Spectra were analysed using Bruker's Compass Data Analysis software. All samples were run using solvent A (0.05% TFA in water) and solvent B (80% acetonitrile, 0.045% TFA in water). Samples were prepared at a concentration of 100 mM peptide in phosphate buffer (20 mM $NaH_2PO_4.2H_2O$, 50 mM NaCl, pH 7.4) and reduced with 5 mM TCEP. Following a 10 minute reduction time the respective compound was added at a 10:1 ratio and allowed to react for two hours. A total of 5 mL of the sample was then loaded into the LCMS.

**pGEL01 sequence design:**

MHHHHHHGKPIPNPLLGLDSTENLYFQGIDPFTGCGGGGSGGGGSGGGGSGSRGHMPPL
TSAQQALTGTINSSMQAVQAAQATLDDFETLPPLGQDAASKAWRKNKMDESKHEIHSQVD
AITAGTASVVNLTAGDPAETDYTAVGSAVTTISSNLTEMSRGVKLLAALLEDEGGNGRPLLQ
AAKGLAGAVSELLRSAQPASAEPRQNLLQAAGNVGQASGELLQQIGESDTDPHFQDVLMQ
LANAVASAAAALVLKAKSVAQRTEDSGLQTQVIAAATQSALSTSQLVASTKVVAPTISSPVS
QEQLVEAGRLVAKAVEGSVSASQAATEDGQLLRGVGAAATAVTQALNELLQHVKAHATGA
GPAGRYDQATDTILTVTENIFSSMGDAGEMVRQARILAQATSDLVNAIKADAEGESDLENSR
KLLSAAKILADATAKMVEAAKGAAAHPDSEEQQQRLREAAEGLRMATNAAAQNAIKKGTGG
GGSGGGGSGGGGS**C**

Red = adaptions; Grey = expression tagged removed in production; C = cysteine attachment sites for attachment of the crosslinker.

**Protein expression and purification:** pGEL, GFP-VD1, mutated_R1, mutated_R2, and GFP were produced as codon optimized synthetic genes in pET151 plasmids (GeneArt), and transformed into BL21(DE3) cells. Overnight cultures were used to inoculate Lysogeny Broth (pH 7.2) or M9 minimal media with $^{15}$N-ammonium chloride for labelled samples, and grown at 37°C until an $OD_{600}$ 0.6-0.8 was reached. The culture was then induced with 100 mM IPTG and grown overnight at 20°C. Harvested cells were resuspended in nickel Buffer A (20 mM Tris pH 8.0, 500 mM NaCl, 20 mM imidazole), sonicated and centrifuged (48,400 x g, 30 minutes, 4°C) . The supernatant was then loaded onto a HP HisTrap Nickel column (Cytiva) connected to an AKTA start system (Cytiva) and eluted with nickel buffer B (20 mM Tris pH 8.0, 500 mM NaCl, 500 mM imidazole). Resulting pure protein was dialysed (10 kDa MWCO) into phosphate buffer (20 mM $NaH_2PO_4.2H_2O$, 50 mM NaCl, pH 7.4) overnight, ready for use. Labelled samples were TEV cleaved following HisTrap Nickel column and dialysed into Q buffer A (20 mM Tris pH 8.0, 50 mM NaCl). The resulting dialysed sample was loaded onto a HiTrap Q HP column connected to an AKTA start system (Cytiva) and eluted with Q buffer B (20 mM Tris pH 8.0, 1M NaCl). Resulting pure protein was dialysed into phosphate buffer (20 mM $NaH_2PO_4.2H_2O$, 50 mM NaCl, pH 7.4) overnight, ready for use.

**Circular Dichroism:** All circular dichroism (CD) experiments were performed on a JASCO J-175 spectropolarimeter using a 1 mm pathlength quartz cuvette. Far UV-spectra were obtained between 200-260 nm with an average of 4 scans at 100 nm/min, 0.5 nm step resolution, 1.0 second response and 0.5 nm bandwidth. For temperature scans CD wavelength was set to 222 nm. Measurements were taken between 20-90°C with 20 second step resolution, 4 seconds of response and 1.0 nm bandwidth. Samples were prepared between 20-50 mM in 400 mL of phosphate buffer (20 mM $NaH_2PO_4.2H_2O$, 50 mM NaCl, pH 7.4).

**Protein NMR experiments:** Protein NMR experiments were conducted at 298 K using a Bruker AVANCE III spectrometer equipped with a QCI-P cryoprobe. $^{15}$N-labelled proteins were measured at 150 mM in 20 mM phosphate, 50 mM NaCl, 2 mM DTT and 5% v/v $^2H_2O$ at pH 6.5. Spectra were processed with TopSpin and CcpNmr Analysis 2.5.2.

**Gel Filtration:** Gel filtration was performed at room temperature using a Superdex 200 Increase size exclusion column (GE healthcare) system at a flow rate of 0.75 mL/min. Samples were run at 150 mM, or at 450 mM for the 3:1 condition, all at final volumes of 100 mL in 20 mM Tris pH 8.0, 150 mM NaCl, 2 mM DTT.

**Light Microscopy:** Images were captured using a Leica light microscope m80 operating at 60x magnification. Samples were removed from the light gas gun after impact exposure and

placed on a glass cover slide and viewed at maximum magnification. Images were then captured by an iPhone SE generation 2.

**Infrared (IR) secondary structure protein analysis:** IR data was recorded on a Shimadzu IR-Affinity-1 model Infrared spectrometer. The data was measured in wavenumbers (cm) against % transmittance using IRsolution software. Data was normalised using a buffer control with or without compound **2** as appropriate before peak deconvolution was performed using OriginPro 8.5 software and multiple peak fitting, manual re-calculate, gauss peak type (4 peaks) data analysis settings. Signal assignment was made using published work by Jackson and Mantsch.[25]

**Estimation of the chain unfolding timescale of the talin domains.** The unfolding rates of talin domains vary according to Bell's model, i.e. the unfolding rate increases exponentially as force increases linearly (k0*exp(f*dx/kT). Each talin domain has a different mechanical stability, with the strongest talin domains unfolding at ~25 pN[18]. Therefore, according to Bell's model all talin unfolding events occur in the picosecond range[18], with the typical relaxation time of peptides in nanoseconds[40].

Estimating the length of the talin protein at 60 nm, a projectile shot at 1.5 km/s will take ~40 picoseconds to pass the length of the protein strand, this is approximately one order of magnitude greater than the protein unfolding time. Applying a basic strain rate estimate, as demonstrated previously by Price *et al.*[41], the strain rate is roughly 1E4 times lower than the reaction speed of the Talin monomer unfolding events. Therefore, for the regime we are operating at, the response of the TSAM is likely to be strain rate independent.

## Supplementary Figures

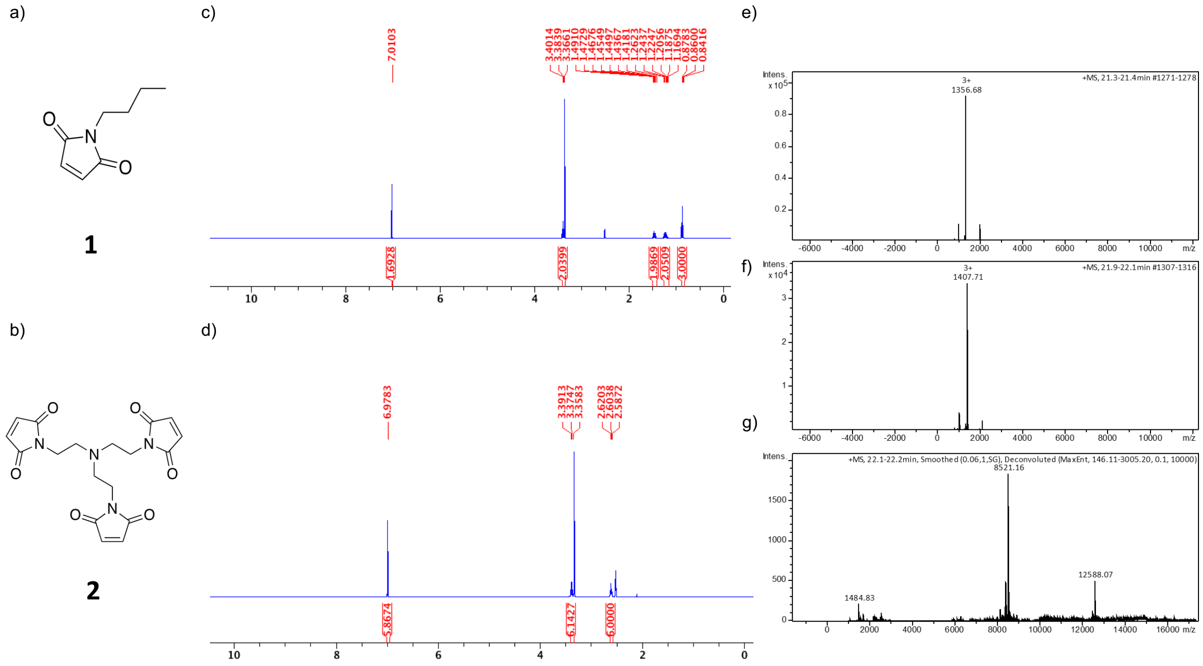


**Supplementary Fig. 1 Compound characterisation.** Chemical structures of compounds **a. 1** and **b. 2. c.** $^{1}$H NMR spectra of compound **1** in DMSO-$d_6$ conducted at 298.15 K, **d.** $^{1}$H NMR spectra of crosslinker **2** in DMSO-$d_6$ conducted at 298.15 K. **e-g.** Liquid chromatography–mass spectrometry (LC-MS) characterisation of crosslinkers **1** and **2**. **e**. KANK1 peptide[38] (95% purity) (0.10 mM, 4067.01 g/mol) used for crosslinking characterisation. **f**. Compound **1** (1.00 mM, 153.08 g/mol) bound to a single KANK1 peptide (0.10 mM) confirming the maleimide group is capable of binding biological macromolecules. **g**. Compound **2** (1.00 mM, 386.12 g/mol) bound to three KANK1 peptides (0.10 mM) confirming all three maleimide groups are capable of binding biological macromolecules.

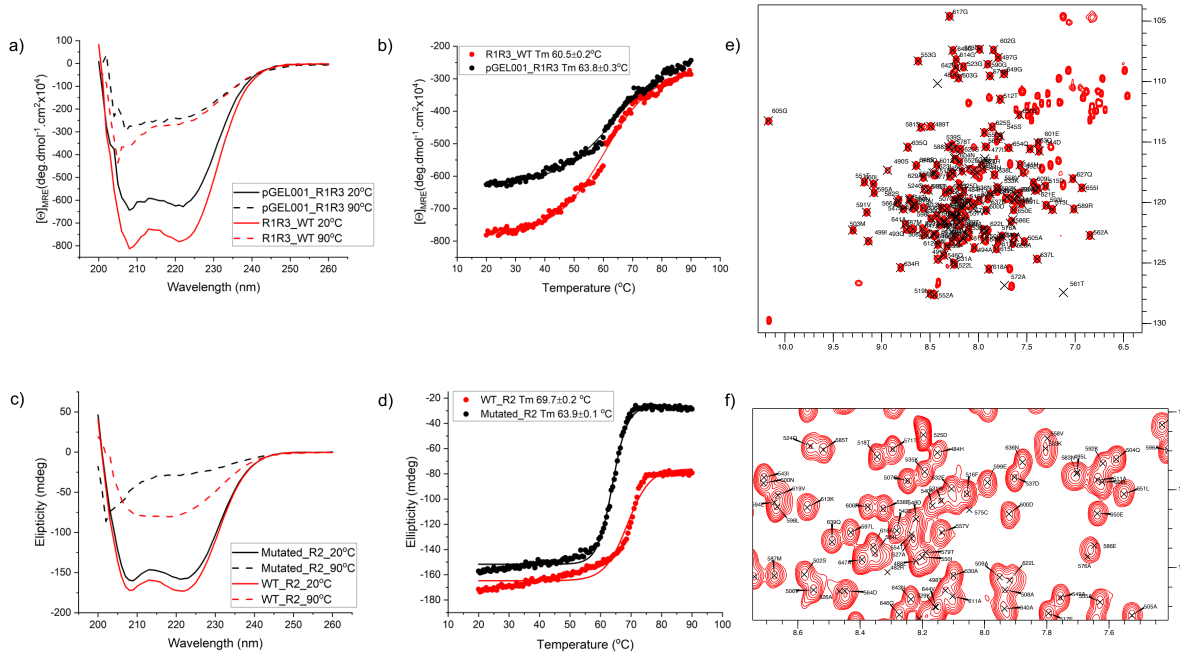


**Supplementary Fig. 2 pGEL characterisation. a-b.** Circular Dichroism analysis of pGEL and wildtype WT-R1R3. **a**. The CD spectra confirm that alpha helical folding is retained in pGEL similar to the wild type R1R3 following the cysteine-serine mutations (1 in R1 and 4 in R2). **b**. The thermal melt curves of the two confirm that only small changes in $T_m$ are observed between the two proteins. **c-d.** Circular Dichroism showing **c.** Alpha helical folding is retained in the mutated R2 after four cysteine residues are mutated to serine. **d**. A change in $T_m$ is seen between the mutated R2 and wild type R2, suggesting minor alterations to stability has occurred. **e.** $^{1}$H,$^{15}$N HSQC spectra of $^{15}$N-labelled mutated R1 with wild type R1 assignments from Banno *et al.*[39] overlaid, showing that the single mutation in the mutated R1 does not perturb the folding of the domain. **f.** HSQC spectra of $^{15}$N-labelled mutated R1 with wild type R1 assignments from Banno *et al.*[39] overlaid, centred on the region where the cysteine in wild type R1 is positioned.

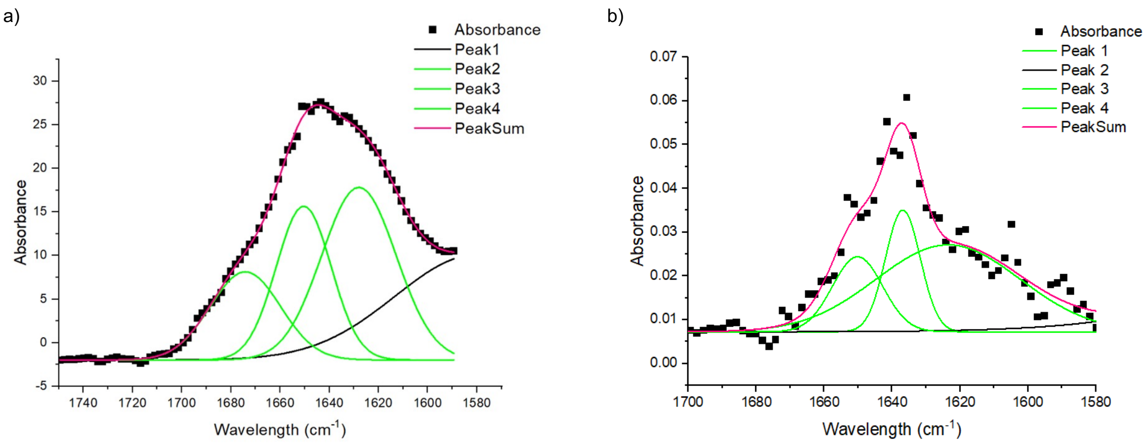


**Supplementary Fig. 3 TSAM characterisation. a-b.** Fourier Transform Infrared (FT-IR) spectroscopy secondary structure protein analysis of hydrated TSAM. **a.** Results from FT-IR analysis of TSAM material prepared in its native state (200 mg/mL pGEL monomer). Black squares = raw data; Green or Black lines = deconvoluted raw data; Pink line = sum of deconvoluted data. Deconvoluted amide I peak maxima (green) identified at 1628 $cm^{-1}$ (possible evidence for the presence of aggregated strands), 1650 $cm^{-1}$ (corresponding to the presence of α-helix structures) and 1674 $cm^{-1}$ (possible evidence for the presence of aggregated strands). Deconvoluted data corresponding to the amide II signal is shown in black and identified as Peak1 within the graph. **b.** Results from FT-IR analysis of pGEL monomer (5 mg/mL). Black squares = raw data; Green or Black lines = deconvoluted raw data; Pink line = sum of deconvoluted data. Deconvoluted amide I peak maxima (green) identified at 1623 $cm^{-1}$ (possible evidence for the presence of aggregated strands), 1637 $cm^{-1}$ (providing evidence for the presence of unordered structures) and 1650 $cm^{-1}$ (corresponding to the presence of α-helix structures). Deconvoluted data corresponding to the amide II signal is shown in black and identified as Peak1 within the graph.

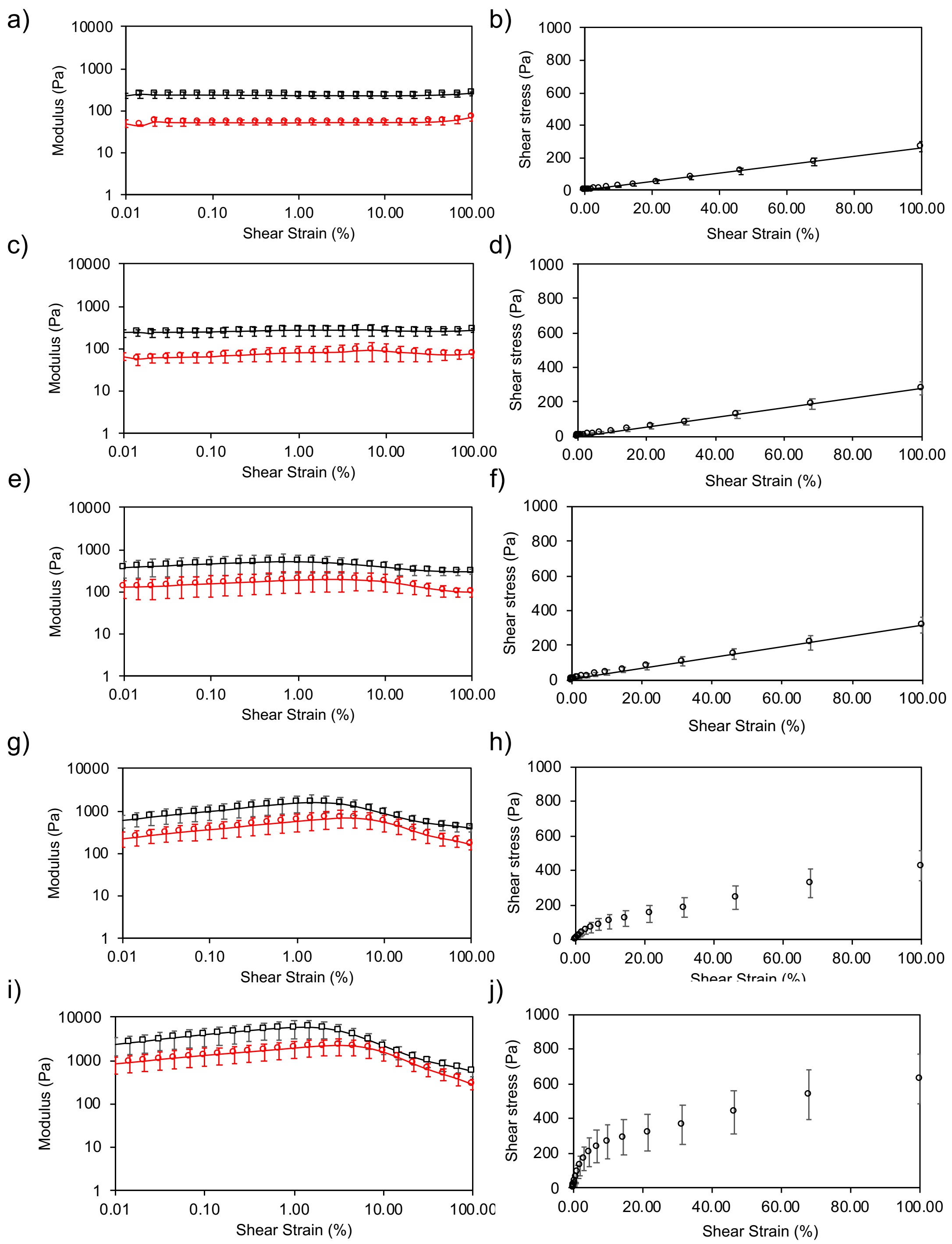


**Supplementary Fig. 4 TSAM rheology.** Rheology data for five amplitude sweeps. **a-b.** Amplitude sweep 1 on TSAM (n = 3, error bars = SEM). **a.** G’ (black) and G” (red) against shear strain and **b.** shear stress against shear strain. **c-d.** Amplitude sweep 2 on TSAM (n = 3, error bars = SEM). **c.** G’ (black) and G” (red) against shear strain and **d.** shear stress against shear strain. **e-f.** Amplitude sweep 3 on TSAM (n = 3, error bars = SEM). **e.** G’ (black) and G” (red) against shear strain and **f.** shear stress against shear strain. **g-h.** Amplitude sweep 4 on TSAM (n = 3, error bars = SEM). **g.** G’ (black) and G” (red) against shear strain and **h.** shear stress against shear strain. **i-j.** Amplitude sweep 5 on TSAM (n = 3, error bars = SEM). **i.** G’ (black) and G” (red) against shear strain and **j.** shear stress against shear strain.

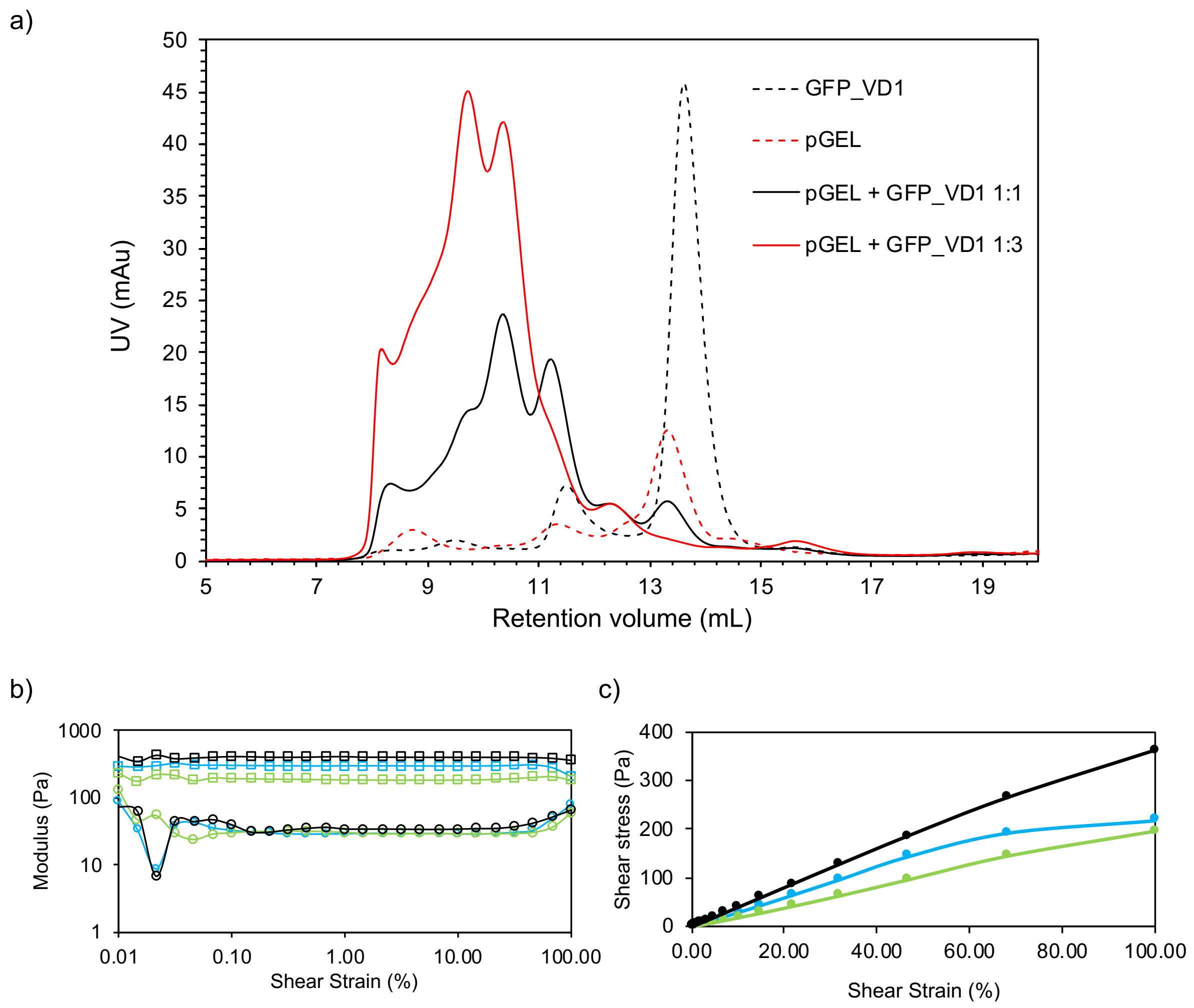


**Supplementary Fig. 5 Analysis of TSAM with GFP-VD1, GFP and buffer. a.** Gel filtration profiles confirming GFP-VD1 binds to pGEL. GFP-VD1 = dotted black line, pGEL = dotted red line, GFP-VD1 + pGEL at 1:1 = solid black line, GFP-VD1 + pGEL at 3:1 = solid red line. **b-c.** Amplitude sweeps from rheological characterisation of TSAM after treatment with phosphate buffer (black), GFP (green) or GFP-VD1 (blue). **b.** G' and G" against shear strain and **c.** shear stress against shear strain for sweep 1.

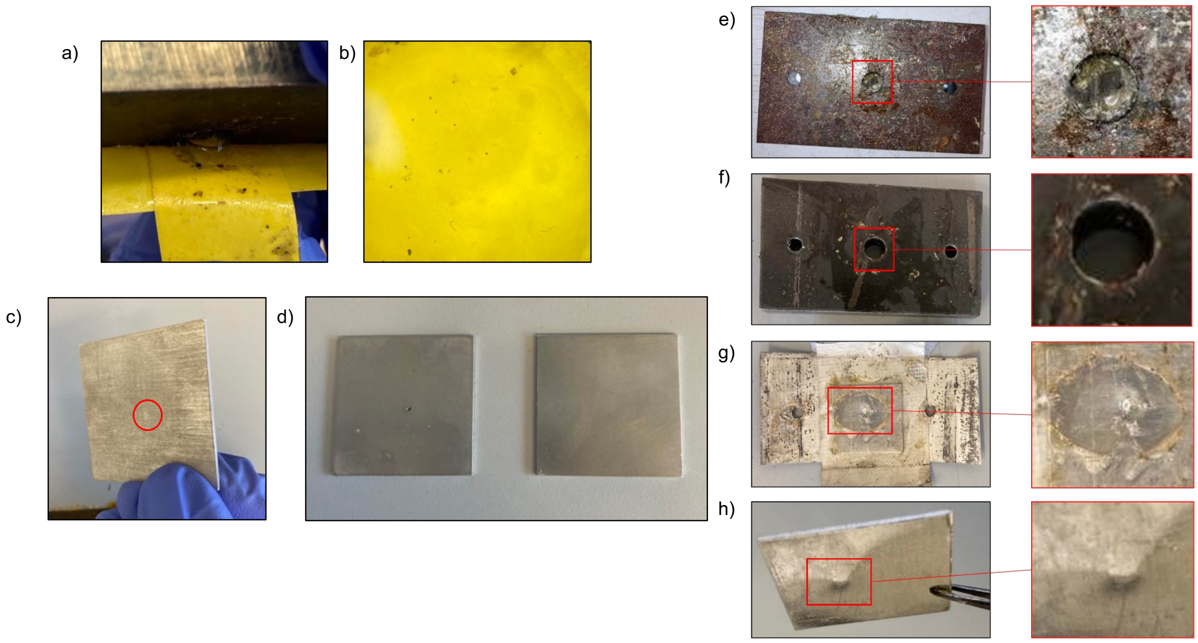


**Supplementary Fig. 6 Supporting images from the Light Gas Gun (LGG) experiments (main manuscript Figure 4). a**. Intact TSAM attached to tape on the back of the BTEA. **b**. Image under light microscope of basalt particles integrated into the TSAM shown in a. **c**. Resulting dent on the back of the aluminium back plate from the control LGG shot. **d**. side by side comparison of the back plates from the control LGG shot (left) and the TSAM LGG shot (right). **e**. Intact TSAM contained within holder after impact at 1.5 km/s. **f**. Destroyed control gel sample after impact at 1.5 km/s. **g**. Perforated backing tape from control sample shot at 1.5 km/s **h**. Resulting dent on the back of the aluminium back plate from the control LGG shot.

a)

b)

c)

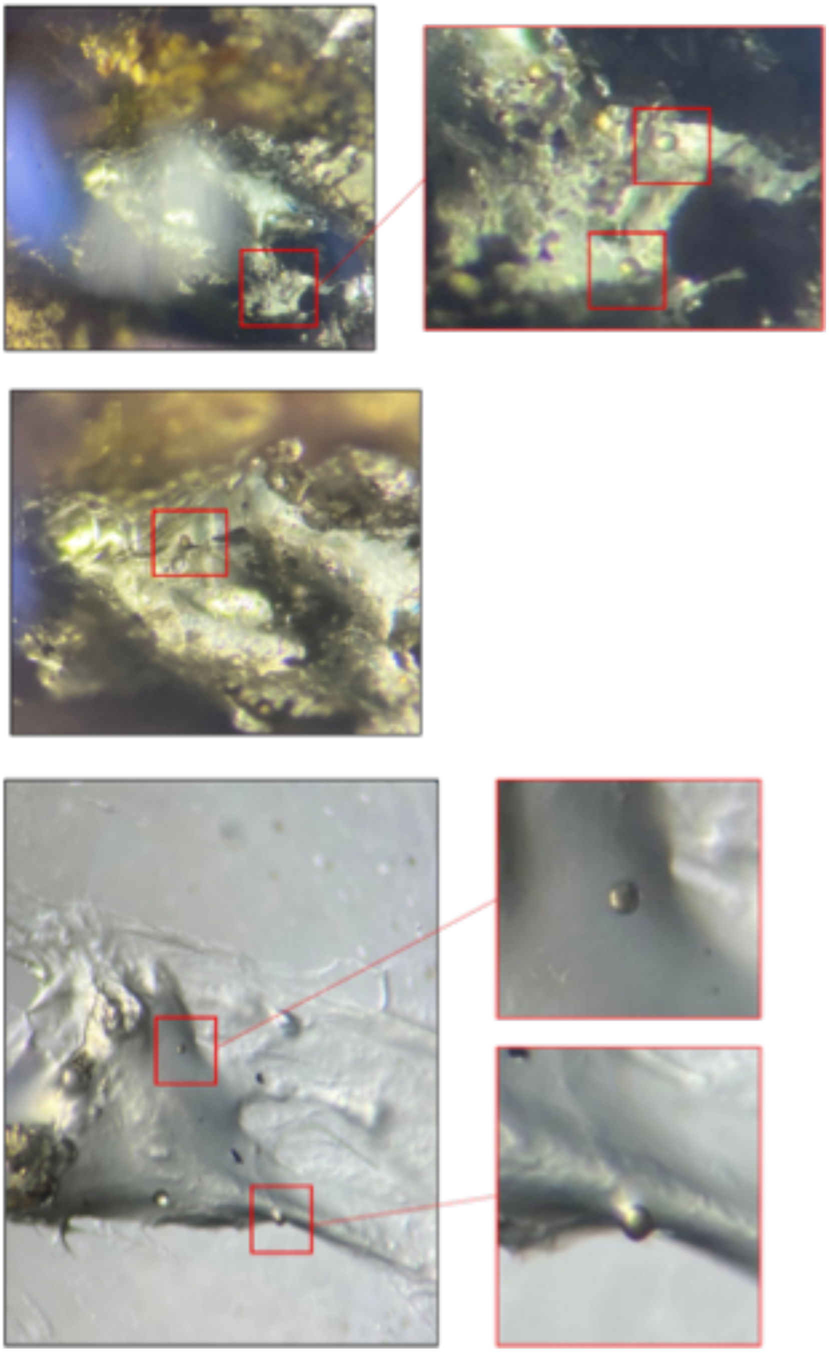

**Supplementary Fig. 7 Supporting images from the Light Gas Gun (LGG) experiments (main manuscript Figure 4). a-c)** Images under light microscope of basalt particles integrated into the TSAM after shot at 1.5 km/s.

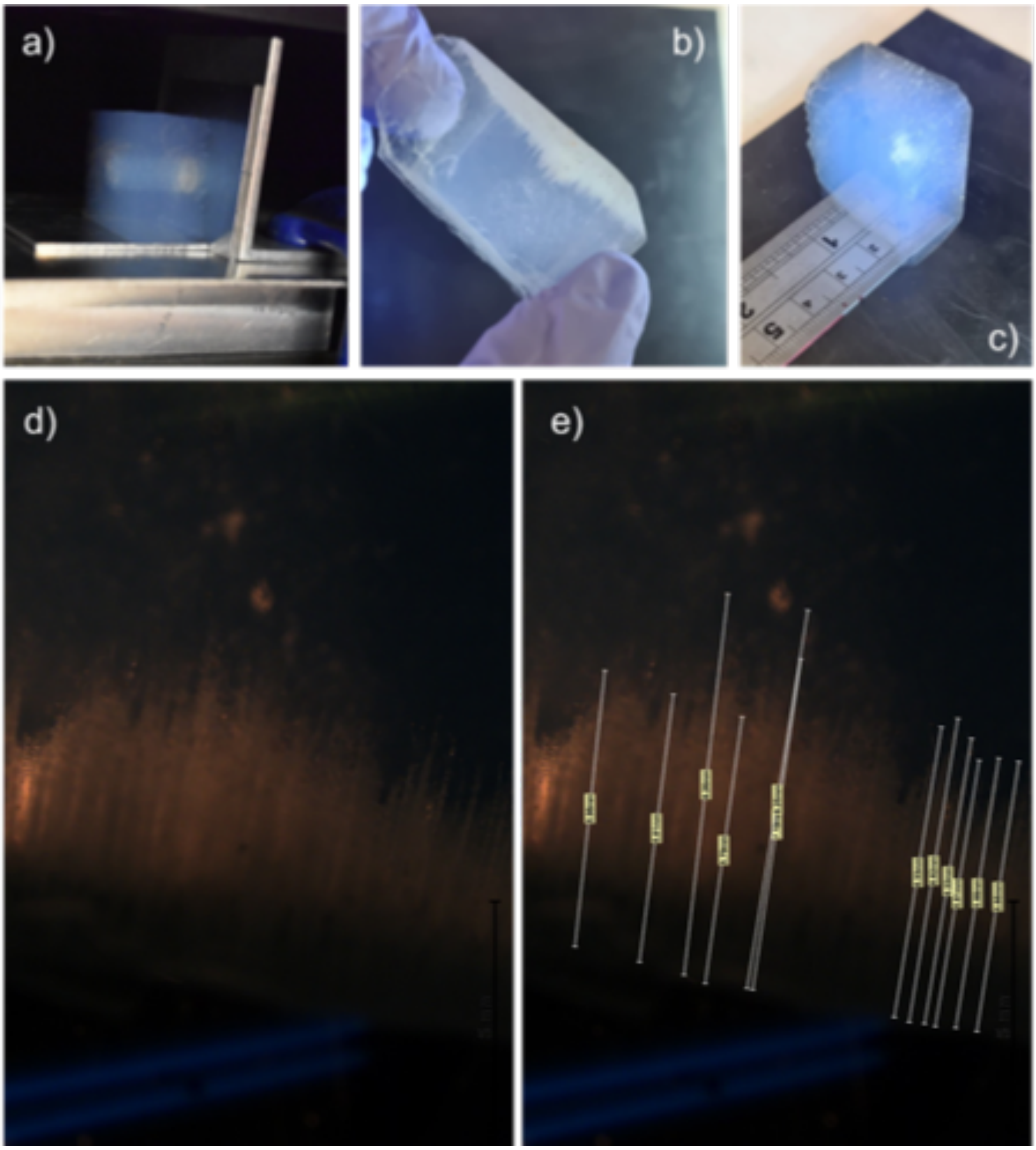


**Supplementary Fig. 8 Supporting images from the Light Gas Gun (LGG) experiments using Aerogel. a**. Aerogel block mounted in the blast tank of the LGG. Note the light beam showing the area of the block to be directly impacted. **b-e**. Aerogel block after impact. A number of tracks can be seen traveling from the surface to a depth of roughly 5 – 8 mm. The entire surface of the target appear ‘sandblasted’ due to impacts from the basalt projectiles fired at 1.5 km/s.

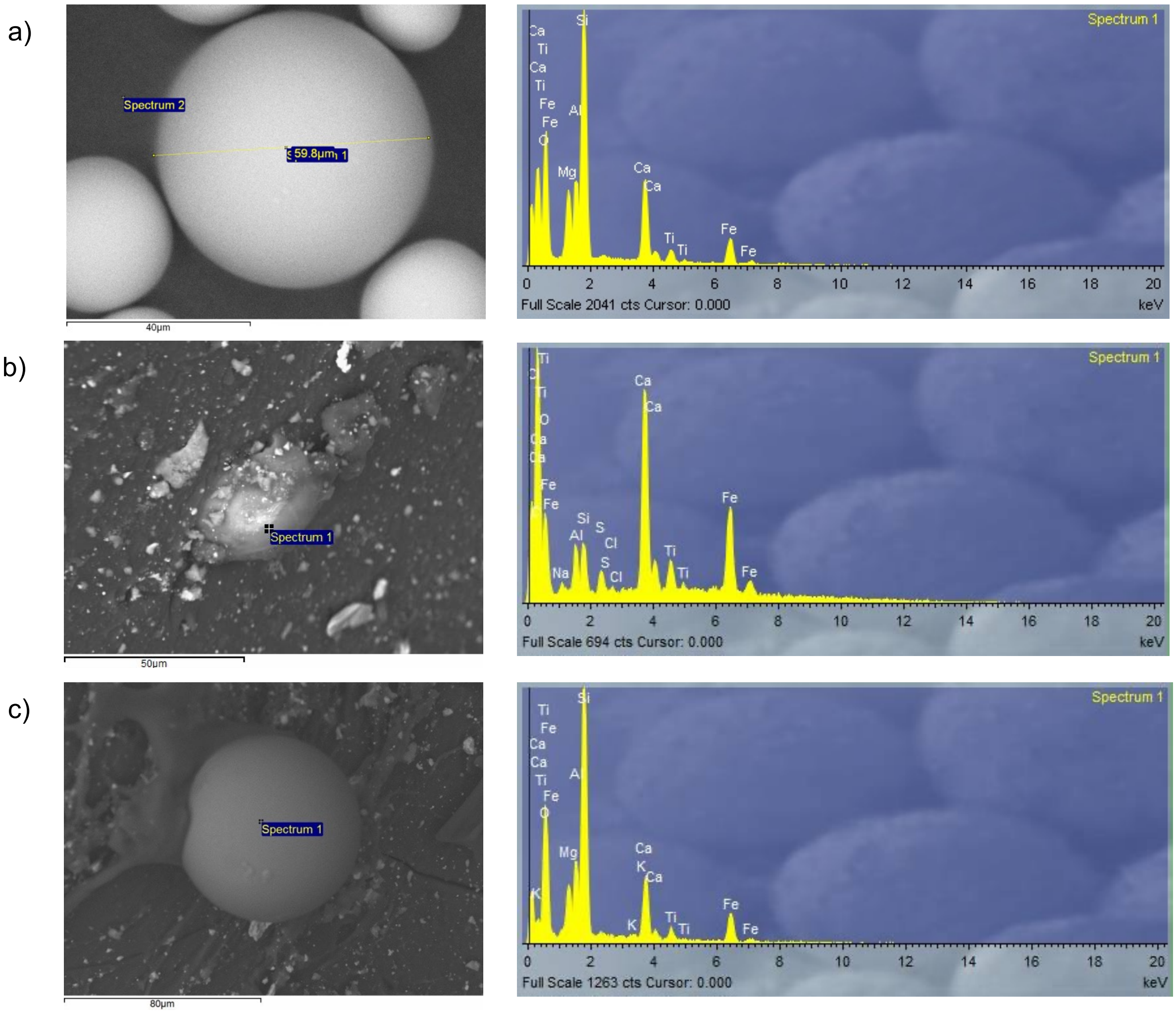


**Supplementary Fig. 9 EDX analysis of caught basalt particles from light gas gun experiments imaged in SEM.** Scanning Electron Microscopy (SEM) images of basalt particles and corresponding elemental dispersive X-ray (EDX) analysis. **a**. Basalt particle before being shot from LGG and its corresponding EDX analysis. **b**. Basalt particle 1 and its corresponding EDX analysis. **c**. Basalt particle 2 and its corresponding EDX analysis. The differences between the EDX spectra shown in panel's a and c in comparison to panel b is due to the presence of gas gun debris, which are created as an experimental by-product.

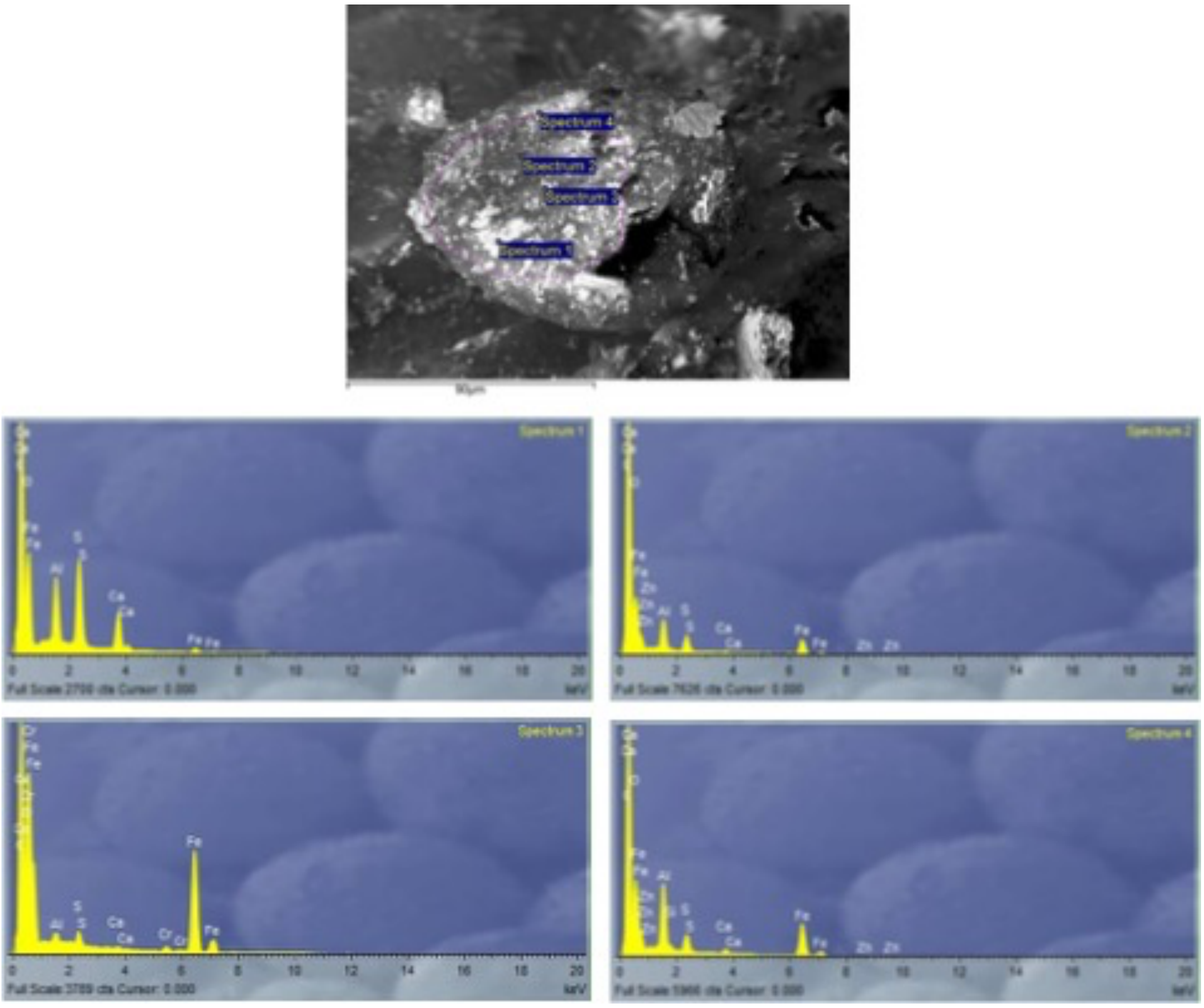


**Supplementary Fig. 10 EDX analysis of caught burst disc shrapnel in light gas gun experiment imaged with SEM.** SEM images of burst disk aluminium fragment and corresponding elemental dispersive X-ray analysis confirming aluminium.

## Methods-only references


18. Yao, M.X. et al. The mechanical response of talin. *Nature Communications* **7**, 11966 (2016).

25. Jackson, M. & Mantsch, H.H. THE USE AND MISUSE OF FTIR SPECTROSCOPY IN THE DETERMINATION OF PROTEIN-STRUCTURE. *Critical Reviews in Biochemistry and Molecular Biology* **30**, 95-120 (1995).

36. Eloh, K. et al. Potent Nematicidal Activity of Maleimide Derivatives on Meloidogyne incognita. *Journal of Agricultural and Food Chemistry* **64**, 4876-4881 (2016).

37. Hanlon, A.M. et al. Exploring structural effects in single-chain "folding" mediated by intramolecular thermal Diels-Alder chemistry. *Polymer Chemistry* **8**, 5120-5128 (2017).

38. Bouchet, B.P. et al. Talin-KANK1 interaction controls the recruitment of cortical microtubule stabilizing complexes to focal adhesions. *Elife* **5**, e18124 (2016).

39. Banno, A. et al. Subcellular Localization of Talin Is Regulated by Inter-domain Interactions. *Journal of Biological Chemistry* **287**, 13799-13812 (2012).

40. Stirnemann G. et al. Elasticity, structure, and relaxation of extended proteins under force. *PNAS*, **110**(10), 3847-3852 (2013)

41. Price, M.C. et al. Validation of the Preston–Tonks–Wallace strength model at strain rates approaching ~$10^{11}$ $s^{-1}$ for Al-1100, tantalum and copper using hypervelocity impact crater morphologies. *IJIE* **52**, 1-10 (2013)
